# Feasibility and preliminary effects of a socio-spiritual intervention for adults with cancer and their family caregivers: a pilot randomised controlled trial

**DOI:** 10.3332/ecancer.2025.1851

**Published:** 2025-02-18

**Authors:** Israel O Gabriel, Debra K Creedy, Amanda McGuire, Elisabeth Coyne

**Affiliations:** 1 Institute of Health and Management, Sydney, NSW, 2150, Australia; 2 Griffith University, Brisbane, Queensland, 4131, Australia; 3 Griffith University, Gold Coast, Queensland, 4215, Australia; a https://orcid.org/0000-0002-5663-450X; b https://orcid.org/0000-0002-3046-4143; c https://orcid.org/0000-0003-3322-285X; d https://orcid.org/0000-0001-8511-600X

**Keywords:** feasibility, effects, socio-spiritual, intervention, unmet needs, quality of life, cancer patient, family caregiver, informal caregiver

## Abstract

**Background:**

Despite significant psychosocial-spiritual needs adversely affecting the health-related quality of life (HRQoL) of adults living with cancer and their family caregivers in sub-Saharan Africa, there is a dearth of culturally tailored interventions to address these needs. This study evaluates the feasibility of a socio-spiritual intervention designed for adults with cancer and their family caregivers in sub-Saharan Africa, and preliminarily examines its impact on family/social support, spiritual needs, information needs, health literacy and HRQoL.

**Methods:**

This study employed a single-site randomised controlled trial design. Eighty-eight dyads were randomly assigned to either a socio-spiritual intervention (*n* = 44 dyads) or usual care (*n* = 44 dyads). The intervention group participated in a 4-week face-to-face training programme with usual care, whereas the control group received only usual care over the same period.

**Result:**

A total of 82 dyads completed the study (40 dyads in the intervention group and 42 dyads in the control group). The eligibility and acceptance rates were >85%, retention was >90% and treatment fidelity was high (between 88.5% and 94.6%). In terms of intervention effects, the intervention was helpful in reducing needs and improving HRQoL of adults with cancer (*F* (13, 65) = 24.50, *p* < 0.001; Wilks' Lambda = 0.17) and their family caregivers (*F* (13, 65) = 14.27, *p* < 0.001; Wilks' Lambda = 0.26).

**Conclusion:**

This pilot study established the feasibility of a face-to-face training programme for adults with cancer and their families, as well as its potential for improving HRQoL of this population.

This study's findings imply that by supporting individuals with cancer and their family caregivers as a unit of care, both individual needs and components of HRQoL can be maintained or even improved. In current practice in Nigeria, only patients' needs are routinely addressed; caregivers often are left on their own to obtain information and support to deliver complex care in the home.

## Introduction

Africa has one of the world's lowest cancer rates; however, this is changing due to an aging population and risk behaviours such as poor eating habits, inactivity, increased alcohol consumption and tobacco smoking [1]. As such, the World Health Organisation predicts that cancer incidence and mortality rates will increase considerably in low- and middle-income countries (LMICs), which include sub-Saharan Africa [2]. Currently in Africa, the death rate due to cancer (7.3%) is greater than the incidence (5.8%) due to a lack of diagnostic and treatment services, poor cancer care, a lack of community resources, a delay in seeking care and African beliefs [3]. In many African societies, cancer is attributed to spiritual rather than physical causes. Cancer symptoms are attributed to a curse, witchcraft or God's retribution for personal or familial wrongdoings or demons, discouraging victims and families from seeking orthodox care in favour of traditional remedies [4].

Despite breakthroughs in cancer therapy, most African countries are still unable to manage prevalent cancers [5]. For instance, Nigeria, Africa's most populous country with over 200 million people, lacks the capability to treat cancer due to insufficient medical facilities, corruption, poor infrastructure and few qualified workers [6, 7]. Many individuals living with cancer may rely on close family members who may also have no or limited resources [8]. Hence, a cancer diagnosis has a profound impact on the patient and family caregivers [9, 10] and is known to affect health-related quality of life (HRQoL) [11–13]. People living with cancer may develop heightened psychological [14], physical [15], social [16] and spiritual concerns [17]. While individuals with cancer face numerous symptoms and uncertainties, family caregivers must balance the demands of caring for their loved ones and addressing their own needs. Both the cancer sufferer and family caregiver undergo significant long-term changes during the cancer trajectory.

Most studies on HRQoL have been conducted in high-income countries with relatively little research evaluating interventions in LMICs despite identified need. For example, Jang and Jeong [18] investigated the relationship between unmet needs and HRQoL in 115 cancer patient-family dyads in South Korea and found a high level of diverse unmet needs negatively impacting both physical and mental components of HRQoL. The authors recommended that intervention programs must target both the needs of patients and their families to improve HRQoL. Another South Korean study with family caregivers of cancer patients found a positive correlation between needs and distress and a negative correlation between needs and HRQoL [19, 20].

A survey of 120 adult Nigerians with cancer and their family caregivers found high social, spiritual and information needs, as well as low health literacy impacted negatively on HRQoL [21]. Sarki and Roni [22] found that Nigerians hold widespread myths and misconceptions about cancer. There is a scarcity of research in Africa and few or no family-based interventions targeting low-income countries. We argue that existing interventions designed and tested in high-income countries are not transferable to the African context. Interventions offered in Africa need to address family, spiritual and cultural issues, all of which are important determinants of HRQoL. This study aims to address these needs by testing a health program that aims to facilitate family support, meet social, spiritual and information needs and promote cancer health literacy (CHLT). The ‘Zaman Lafiya Program’, is a socio-spiritual intervention that is the local vernacular term for ‘Living Well’.

The specific aims of the study were to (1) test the feasibility of a socio-spiritual intervention suitable for the clinical realities of sub-Saharan Africa (primary outcome) and (2) evaluate the effectiveness of the socio-spiritual intervention on family/social support, spiritual and information needs, health literacy and HRQoL (secondary outcomes).

It was hypothesised that adults with cancer and family caregiver dyads who received the intervention would have significantly higher HRQoL, family/social support, CHLT and lower spiritual and information needs than the control group.

## Methods

### Study design and participants

The study was designed as a pilot randomised controlled trial (RCT). It is part of a larger research project with the full trial protocol reported elsewhere [23]. The trial was registered with the Pan African Clinical Trial Registry under the identifier PACTR202007829295775. The reporting of this study adheres to the Consolidated Standards of Reporting Trials (CONSORT) [24].

Adults with a confirmed cancer diagnosis within the last 3 months were eligible for the trial together with their family caregivers (dyads). All participants were recruited from an outpatient oncology clinic of the Ahmadu Bello University Teaching Hospital in Nigeria. Participants were at least 18 years old, mentally and physically capable of participating, literate to the junior secondary school level (Grade 9) and able to find a willing family caregiver. Primary family caregivers were 18 years old or older, mentally and physically capable of participating, speak and understand English and designated by the person with cancer. The study excluded adults with cancer but no family caregiver, those who could not read or write in English, skilled healthcare providers who have cancer or care for people with cancer and family caregivers receiving treatment for a disease that hinders their physical or psychological well-being.

A power analysis determined the sample size required to evaluate the intervention. According to Northouse *et al.* [25], a sample size of 38 dyads in each arm would give 85% power for repeated-measures analysis of variance with a moderate effect size and probability of 0.05. Additional recruitment of 15% was made to allow for attrition.

### Randomisation

Following recruitment, participants were assigned to either the intervention group (usual care plus the intervention programme) or control group (usual care only) through simple randomisation. The randomisation sequence was established through computer-generated block randomisation in a 1:1 ratio to ensure equitable distribution among the groups. To uphold the integrity of the process, a number of essential measures were implemented. Initially, participants remained uninformed about their group allocation following recruitment yet before randomisation, thereby maintaining impartiality. Second, the allocation was carried out by an independent researcher who was not involved in recruitment or data collection, thereby ensuring a completely unbiased and concealed process. In addition, the details of the randomisation were concealed from the recruitment team, thereby strengthening the blinding process. To mitigate any response bias, all participants, regardless of their group assignment, received questionnaires with uniform phrasing and timing. The rigorous randomisation processes were designed to enhance the study's internal validity and the reliability of the results, despite the interactive nature of the intervention preventing the blinding of participants and instructors.

## Procedure

### Establishment of the research team

A research team was formed, combining academic knowledge and practical experience to create a comprehensive and fully integrated programme. The team consists of two certified nurse educators with a substantial background in oncology nursing and research methodologies, as well as three registered nurses possessing extensive clinical oncology experience. The team received 5 days of training on the study aims, procedures, surveys, content of four sessions, group processes, use of registration forms and data collection, during which they became acquainted with the programme and their position within it. In addition, the team received a digital version of the intervention protocol and facilitator guide. The significance of rigour was emphasised.

### Programme development

The researchers developed a theory-based, culturally tailored socio-spiritual intervention to address the specific needs of adults living with cancer and their family caregivers, based on systematic literature review findings [26] and needs assessment and HRQoL [21].

The frameworks of the programme were derived from four main sources: Medical Research Council framework for developing and evaluating complex interventions [27], the Behaviour Change Wheel [28, 29] to understand and define target behaviours, Hodge [30] Spirituality and Supportive Care Framework for Cancer Care [31]. These frameworks were used to develop an evidence-based socio-spiritual intervention (referred to as the ‘Zaman Lafiya Programme’, a local vernacular name that means ‘Living Well’).

The programme progresses through a three-stage developmental pathway: first, identifying the evidence base through a systematic review and needs assessment; second, identifying and developing theory, which encompasses identifying theories, methodologies, modes of delivery, operationalising findings into intervention components; and third, modelling process and outcomes, incorporating findings from a stakeholder expert panel [32]. This intervention development has been published elsewhere [32].

The programme content and processes were reviewed by experts (oncology clinicians and researchers) and modified based on their feedback. Revised content was then produced into a booklet for participants and supporting videos developed as resources. The programme comprises four sessions, each lasting 2 hours, focussing on diverse behavioural and social factors influencing HRQoL.

A programme facilitator booklet was developed with detailed information about the themes discussed in each session.

### The intervention group

The intervention group dyads in addition to usual care had a 4-week face-to-face socio-spiritual intervention in addition to usual care. The programme consisted of four main sessions. Session 1 (week 1) concentrated on understanding the cancer journey, needs for family/social support and spirituality. Session 2 (week 2) addressed the topics of sharing the experience, effective communication and building trusting relationships. Session 3 (week 3) addressed support systems, including family, social and spiritual support, while session 4 (week 4) focussed on resilience in the face of adversity, understanding the strengths and resources of the family by building spiritual support. The topics and related content are presented in Table 1.

Each session included a 10-minute video clip featuring simulation/cancer survivors and their families sharing their experiences according to the session topic. The intervention was delivered by two registered nurse educators with extensive experience in oncology nursing and research. Each nurse led two concurrent group sessions in the out-patient department on various days of the week. Each group consisted of five to seven adults with cancer and their caregivers. Group sessions provided an opportunity for participants to express their feelings and discuss issues of shared concern. All participants received the intervention between December 2022 and April 2023. One of the research teams contacted participants by telephone in advance to inform them about the upcoming session and encourage attendance. This approach was intended to be supportive and identify any difficulties participants may be encountering (such as transport and appointment clashes).

At the conclusion of the programme, each study participant got the equivalent of AUD$5 to assist with transportation costs.

### The control group

The dyads within the control were not subjected to any specific interventions throughout the study duration. However, their usual care encompasses routine medical and nursing services provided by healthcare professionals, cancer therapy, medication, counselling and nutritional guidance.

The control group was apprised of the overarching framework of the study and that they would be granted access to the same training programme upon the conclusion of the study period.

### Data collection

**Figure 1.**
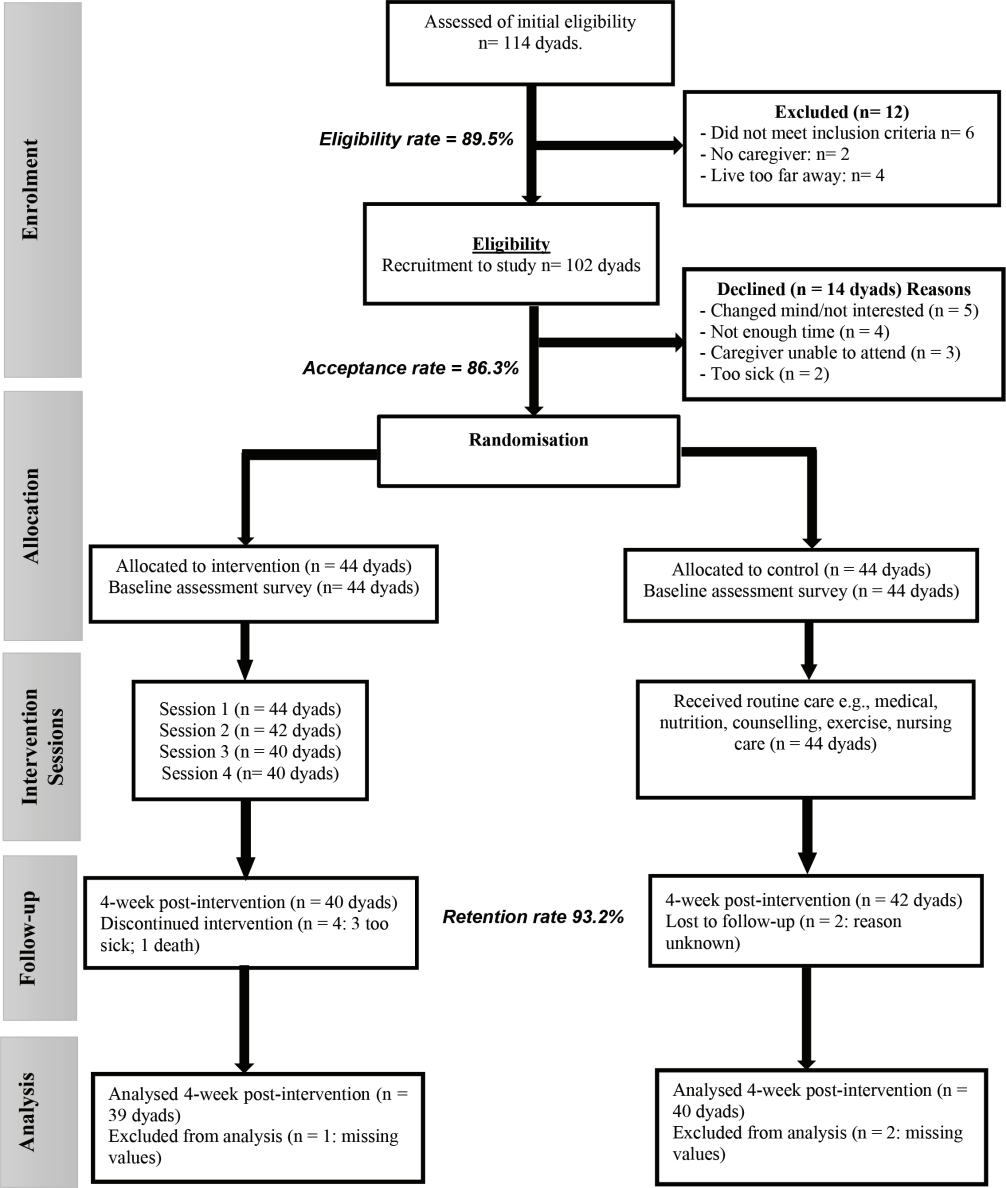
CONSORT participant flow diagram.

Eligible dyads were informed about the study by the health care professionals working in the out-patient department where adults with cancer were being treated. The research team contacted interested dyads and, if willing, provided an information sheet and written informed consent. During this period, the intervention group participated in a 4-week in-person training program, while the control group received no immediate training beyond their usual care. Data collection was conducted by surveys administered at two-time points: baseline (T1) and immediate post-intervention (4-weeks, T2) (Figure 1).

Both the intervention and control groups administered the same surveys at the commencement of the study, encompassing demographic data, the Multidimensional Scale of Perceived Social Support (MSPSS), the Spiritual Needs Assessment for Patients (SNAP), the Comprehensive Needs Assessment Tool for Cancer (CNAT), the Comprehensive Needs Assessment Tool for Cancer-Caregivers (CNAT-C), the CHLT-6 and the Functional Assessment of Chronic Illness Therapy-Spirituality (FACIT-Sp). Following the intervention, both groups were requested to complete the follow-up survey, which mirrored the first questionnaire to guarantee data comparability.

The intervention group offered input regarding the training program's acceptability and engagement levels. The surveys were completed within 20 minutes with the support of the research team.

## Measures

### Demographic information

Data were collected pre-intervention (baseline) and post intervention (4 weeks) by a research team member who did not participate in the delivery of the Zaman Lafiya intervention or the control condition. Participants provided information on their gender, ethnicity, marital status, education, religion, occupation, monthly income, relationship to the adult with cancer, cancer type and stage of cancer.

### Feasibility

Feasibility was measured by (1) acceptance, eligibility and retention rates; and (2) intervention fidelity. A recruitment rate of 60% was considered acceptable [33]. Our retention and assessment benchmarks were set at 80%, which meant that if 80% of dyads completed all sessions and assessments, study feasibility would be demonstrated. Fidelity was determined by evaluating sessions for a subset (50%) of participants using a structured adherence checklist form (see Appendix for sample checklist). Intervention fidelity could range from 0% to 100% depending on the number of session topics covered. Fidelity levels of 80% to 100% were regarded as high [34].

### Family/social support, spiritual need, information need, health literacy and HRQoL

Family/social support was measured using the MSPSS, with higher scores indicating increased support [35]. Reliability and validity have been demonstrated ranging from 0.93 to 0.98 [36].

Spiritual need was measured using the SNAP [37], with higher values indicating greater spiritual needs [38]. SNAP has a Cronbach’s alpha of 0.95 [39].

The CNAT subscale [40] and CNAT-C subscale [41] measured information needs in adults with cancer and family caregivers, with higher scores indicating greater need. The subscales have good internal consistency with Cronbach's alpha coefficients ranging from 0.79 to 0.97 [21].

The CHLT-6 [42] was used to measure participants' CHLT.

The FACIT-Sp [43, 44] measured HRQoL, with higher scores indicating well-being.

### Validity and internal consistency

The included measures had been validated in several countries as well as a cross-sectional sample of Nigerian adults with cancer and their family caregivers (Author blinded, 2021). Cronbach’s alpha ratings for each scale were computed in this study, and internal consistency and reliability were confirmed. The Cronbach’s alpha for the MSPSS ranged from 0.96 to 0.98 for total and subscales, SNAP ranged from 0.62 to 0.96 for total and subscales, CNAT ranged from 0.87 to 0.91, CHLT-6 ranged from 0.61 to 0.89 and FACIT-Sp ranged from 0.66 to 0.92 for total and subscales.

### Data analysis

All statistical analyses were conducted using an intention-to-treat approach. The Statistical Package for Social Sciences (SPSS version 27) [45] was used for the analysis. Means and standard deviations were used to summarise continuous demographic and clinical variables, while frequencies, percentages and 95% confidence intervals were used to describe categorical data. Skewness and kurtosis were performed to determine the distribution normality. There are some skewed and kurtotic data, but they are not statistically different from normality, for both persons living with cancer and family caregivers.

A multivariate approach using analysis of variance (MANOVA) was taken to assess the effects of the intervention on secondary outcomes. This approach was chosen because the effect of the intervention on specific variables (e.g., family/social support, spiritual need, information, CHLT and HRQoL) would provide more direction for future modification and implementation of the intervention.

### Ethical considerations

Approval was granted by the Human Research Ethics Committee of Griffith University, Queensland, Australia (GU Ref No 2020/554) and Ahmadu Bello University Teaching Hospital (ABUTH), Zaria, Nigeria (ABUTHZ/HREC/W38/2020). The research was conducted in accordance with the Helsinki Declaration, which outlines ethical principles for medical research. It was clearly articulated that participants retained the right to withdraw from the study at any moment without facing any repercussions. All data gathered during the study were anonymised to safeguard participant confidentiality and maintain the integrity of the data.

## Results

### Participants characteristics

Table 2 lists demographic and clinical characteristics of participants. Adults with cancer in the final sample were 51.9 years old (SD = 14.8; age range, 18–81), while family caregivers were 41.9 years old (SD = 10.9; age range, 19–64). Over half the participants were female (adults with cancer, 54.4%; family caregivers, 60.8%) and married (>75%). Most had a high school certificate or less. The tribal composition revealed the majority were Hausa (48%). Most participants (74.7% of adults with cancer and 67.1% of family caregivers) earned less than $500 per month. Breast cancer was the most common type of cancer in adults, followed by cervical and lung cancer, and finally prostate cancer, with most patients diagnosed at stage II or III. There were no significant differences in any sociodemographic variables between the intervention and control groups at baseline (Table 2).

### Feasibility

In total, 114 dyads who were approached expressed interest in the study. Of those, 102 dyads (89.5%) met eligibility criteria and were invited to participate. Of the 102 eligible dyads, 14 dyads (13.7%) refused to participate (Figure 1). Eighty-eight dyads consented to the study and were randomly assigned to the intervention or control group (intervention *n* = 44; control *n* = 44) and completed baseline measures, reflecting a high acceptance rate of 86.3%.

Four of the 44 dyads that began the Zaman Lafiya intervention did not complete all sessions as well as the post intervention assessment (4-week time point), and two of the 44 dyads who completed the baseline assessment did not complete the post intervention assessment. Thus, the retention rate was 93.2%, indicating adequate retention and assessment per the 80% a-priori cut‐off as the benchmark for feasibility.

Of the 82 dyads who completed the final survey (40 in the intervention group and 42 in the control group), 79 were used in the analysis; three dyad responses (one in the intervention group and two in the control group) were excluded due to missing data. The error rate was 3.7% (<10 %) (Figure 1). The study attrition rate was not significantly different by group at Time 1 or Time 2.

A research assistant assessed fidelity using a standardised adherence checklist form to evaluate sessions for a subset (50%) of sessions (Supplemental materials for checklist). Based on the topics covered in the session, treatment fidelity ranged between 88.5% and 94.6%, exceeding our fidelity threshold of 80%. There were no adverse events reported during the implementation of the intervention.

### Effects on family/social support, spiritual need, information need, health literacy and HRQoL

Preliminary assumption testing was conducted to check for normality, linearity, univariate, multivariate outliers and multicollinearity, with no serious violations noted. Tables 3 and 4 show means and standard deviations (derived from MANOVA) for all five dependent variables (family/social support, spiritual needs, information needs, CHLT and HRQoL) for adults with cancer and family caregivers in the intervention and control groups. No significant baseline differences between groups were found.

Overall HRQoL and three of its five components (social, functional and spiritual wellbeing), overall family/social support and its subscales (significant other, family and friend subscales), and CHLT scores increased over time (from baseline to 4-week) in both adults with cancer and family caregivers, while spiritual and information needs decreased within the intervention group.

Compared to the control group, mean scores of both adults with cancer and family caregivers in the intervention group on family/social support and its subscales, CHLT, HRQoL and three components (social, functional and spiritual wellbeing) were higher, while spiritual and information needs were lower. Two components of HRQoL (physical and emotional well-being) decreased both within and between groups.

MANOVA revealed a statistically significant effect of group and time interaction on the combined dependent variables for adults with cancer, *F* (13, 65) = 24.50, *p* < 0.001; Wilks’ Lambda = 0.17; and family caregivers, *F* (13, 65) = 14.27, *p* < 0.001; Wilks’ Lambda = 0.26. This finding shows that differences in scores over time could be attributable to the intervention (Tables 3 and 4).

A significant effect of the time x group interaction was observed in each subscale of the dependent variables for adults with cancer, including the significant other, friend subscales, information scale, social wellbeing and functional wellbeing. Similarly, the interaction between time and group had a significant effect on the spiritual subscale, information scale, CHLT, physical, social, functional and spiritual well-being of family caregivers (Tables 3 and 4).

## Discussion

This study successfully evaluated the feasibility and effectiveness of an intervention that addressed the socio-spiritual needs of adults living with cancer and their family caregivers in LMICs and improved their HRQoL. The brief intervention was delivered once a week for four consecutive weeks in a community setting. The study was successful in collecting data to inform a larger roll-out of the intervention in future research. There are currently no interventions targeting the spiritual needs, as well as CHLT, of individuals living with cancer and their families in LMICs, particularly on the African continent. Thus far, interventions in this population have been developed for high-income countries, and most of the content lacks spiritual and health literacy components. The current study is, to the best of our knowledge, the first to design and test an intervention on crucial components of Africans' well-being, such as spiritual and social well-being and CHLT.

### Feasibility

A theoretical and evidence-based approach was used. Findings indicated that the program was feasible to implement, and the intervention group demonstrated statistically significant improvements in family/social support, spiritual needs, information needs, CHLT and HRQoL. The socio-spiritual intervention was well received by participants, as demonstrated by high acceptance and retention rates. A subset (50%) of sessions had treatment fidelity ranging between 88.5% and 94.6%.

When designing this intervention, our panel of experts stressed the importance of feasibility, emphasising the ease with which sessions need to be scheduled (e.g., intervention venue, session length and a number of participants, mode of delivery) and session content organised. The low attrition rate in the intervention arm, as well as the fact that most participants attended all sessions, demonstrate the advantages of this flexible approach, which included conducting two concurrent group sessions in the out-patient department on different days of the week, with group size being contained (between 10 and 14 people) to foster discussion and engagement. In addition, the facilitators were locals who contacted participants via telephone to remind them about the upcoming session, encourage attendance and overcome any practical barriers to attendance.

In future iterations of program delivery, we may include a range of clinicians and test alternative recruitment strategies and elicit qualitative feedback from adults with cancer and their families regarding their preferred approaches. Difficulties enrolling patients and/or family caregivers and high attrition rates have been reported by other researchers, most notably during the COVID-19 lockdown [46]. In this study, however, intrinsic beliefs, the influence of significant others such as health professionals, and modest incentives to assist with transportation costs all served as motivators for participation and adherence to the intervention.

### Effectiveness

The secondary outcome of this study was to determine the effectiveness of the Zaman Lafiya program on HRQoL. According to the findings, the program contributed to increasing family/social support, CHLT and reduced spiritual and information needs, all of which have a positive influence on HRQoL. One notable finding was that adults with cancer who participated in the intervention reported significantly more family/social support after 4 weeks than adults with cancer in the control group. The research literature contains numerous reports indicating that how patients respond to diagnosis and treatment is highly dependent on both professional and family/social support [47, 48]. There are few reports on how to promote family/social support, particularly in African countries where social support is critical for the survival and HRQoL of patients with chronic conditions. Even though the intervention had a significant effect on perceptions of family/social support for adults with cancer, family caregivers reported no change despite participating in all aspects of the intervention. It is possible that the intervention was directed more at improving family/social support in adults with cancer than in family caregivers. Another possibility is that family caregivers are often not seen as valuable resources and who need support themselves to give support [49].

Another important finding from this study was that while adults with cancer in the intervention group reported significantly less information needs, CHLT improved over time but was not significantly. This finding is consistent with an earlier study that investigated the information needs of cancer patients’ (*n* = 104) during radiotherapy and found that low health literacy was associated with decreased information needs [50]. Similarly, Matsuyama* et al.* [51] observed that cancer patients with low health literacy did not have higher information needs. In contrast, [52] reported that cancer patients with low health literacy have higher information needs. Given the considerable reduction in information needs, one may expect CHLT to improve significantly as a result of the intervention content; however, this was not the case. One possible explanation is that ‘information needs’ is a multidimensional construct that constitutes health literacy; as well as medical knowledge, motivation, competence, appraisal, ability to make judgments and make healthcare-related decisions in everyday life [53]. Perceived need for information is a broader concept than health literacy. Another reason may be the development of health literacy, which Kwan *et al.* [54] described as a generative concept that develops along a trajectory toward a number of milestones over a lifetime. Even though the intervention had a significant positive effect on information needs, it may not have influenced immediate changes in health literacy.

Surprisingly, both the information needs and health literacy of family caregivers were significantly improved. This could be because, unlike adults living with cancer, family caregivers were better equipped to appraise the knowledge and information, and had the competence and motivation required to achieve adequate health literacy [55]. Furthermore, in this study, family caregivers were younger and better educated than their cancer-affected family members. This is consistent with the findings of Vamos *et al.* [56], who identified educational attainment and age were predictors of health literacy.

Regarding HRQoL, significant effects were observed overall and two components (social and functional) in cancer patients, as well as overall and four components (physical, social, functional and spiritual) in family caregivers. This is not surprising given that numerous studies have found a negative association between the needs of patients and/or family caregivers and HRQoL [20,21]. Because the adults with cancer were diagnosed within the last 3 months, shock, disappointment and fear of uncertainties may have contributed to the non-significant effect on physical, emotional and spiritual components of HRQoL.

## Limitations

Despite the positive feasibility and effects on dependent variables, there are limitations. First, our sample was entirely Nigerian, and although the intervention targeted this group, sample characteristics may limit generalisability. Second, the heterogeneity of participants in terms of cancer types and stages of cancer might influence outcomes reported on the various measures. Cancer prognosis and treatment progress have an unavoidable impact on participants' well-being. As a result, changes in outcomes measured may be attributed to factors other than the intervention. Another limitation is that the intervention is only available to participants who can speak and write in English. This excludes non-English-speaking cancer patients and family caregivers, who may have different supportive needs.

### Strengths and implications for practice

The single-blind, intent-to-treat, RCT design and use of reliable and valid outcome measures were major strengths of this study. In addition, the socio-spiritual intervention program was theoretically driven and developed in response to the findings of a systematic review and a needs assessment. This enhanced the relevance and applicability of the intervention and possible replication in other studies.

This study has implications for practice because the findings imply that by supporting individuals with cancer and their family caregivers as a unit of care, both individual needs and components of HRQoL can be maintained or even improved. In current practice in Nigeria, only patients' needs are routinely addressed; caregivers often are left on their own to obtain information and support to deliver complex care in the home.

Finally, although this study found significant effects for adults with cancer and their family caregivers, future studies should examine program dose and its effect on intervention outcomes.

## Conclusion

This trial established the feasibility of implementing the Zaman Lafiya program for adults with cancer and their family caregivers in community settings in Nigeria. This novel intervention is unique in that it combines specific needs with well-established theory to develop a targeted intervention for oncology patients. We demonstrate that it is possible to better serve the needs of people living with cancer and their families in LMICs. The intervention warrants a full-scale investigation to validate the findings and assure its long-term validity. Future research should prioritise this type of intervention, with an emphasis on medium- and long-term follow-up to see whether these gains are sustained over time.

## List of abbreviations

ABUTH, Ahmadu Bello University Teaching Hospital; CHLT-6, Cancer health literacy; CNAT, Comprehensive needs assessment tool; CNAT-C, Comprehensive needs assessment tool for cancer caregivers; CONSORT, Consolidated standards of reporting trials; FACIT-Sp, Functional assessment of chronic illness therapy – spiritual well-being scale; HRQoL, Health-related quality of life; LMICs. Low-middle-income countries; MANOVA, Multivariate analysis of variance; MSPSS, Multidimensional scale of perceived social support; SD, Standard deviation; SNAP, Spiritual needs assessment for patient; T1, Time 1; T2, Time 2; RCT, Randomised controlled trial.

## Conflicts of interest

The authors have declared that they have no possible conflicts of interest in connection with the research, authorship or publication of this paper.

## Funding

No funding was obtained.

## Figures and Tables

**Table 1.** Four core components of the Zaman Lafiya program.

Sessions	Core component	Goals	Group processes
Session 1	Understanding cancer journey	To appreciate the experience and common concerns of people affected by cancer across the cancer journey. To describe the strategies employed in the local setting to promote approaches to care.	Help participants share fears and concerns about cancer and its treatment Educate about illness and treatments and caregiving according to identified knowledge gaps and misconceptions based on participant feedback. Encourage optimistic thinking Communicate and demonstrate a range of possible therapies such as relaxation, massage. Encourage healthy coping and lifestyle behaviours
Session 2	Communication and building trusting relationships	To encourage the expression of feelings and ideas. To assess verbal and nonverbal client communication needs. To respect the client's personal values and beliefs.	Ask participants to write down their concerns and fears about cancer prognosis. Discuss results amongst the group. Promote open communication. Encourage mutual support and teamwork. Promote approaches to helping dyad work within the limits of their new limitations.
Session 3	Finding the right support	To provide relief from suffering, manage symptoms, help reconcile relationships, and assist in the transition between this life.	Help dyad to stay hopeful in the face of death through honest conversations with family and close friends. Social connections; link between the past and present. Help dyad deal with overwhelming stress by talking to someone they trust, exercising, and making time for activities they enjoy
Session 4	Understanding strengths and resources of the family building.	To recognise family’s positive attributes. To helps families cope during times of trouble.	Identify family strengths Use reflective writing to deepen insights, reflect on life changes and what is essential in life. Discuss the use of spiritual coping with health challenges Consider what provides a sense of inner peace for the individual. Explore the views of each other and come to a new shared understanding.

**Table 2.** Demographic and clinical characteristics of participants.

Variable	All samples (*N* = 79 dyads)		Intervention group (*N* = 39 dyads)		Control group (*N* = 40 dyads)		Intervention and control (*p*-value)	
	Adults with cancer *N* (%)	Family caregivers *N* (%)	Adults with cancer *N* (%)	Family caregivers *N* (%)	Adults with cancer *N* (%)	Family caregivers *N* (%)	Adults with cancer	Family caregivers
Mean age (SD)	51.9 (14.8)	41.9 (10.9)	52.7 (15.9)	41.9 (11.0)	51.0 (13.8)	41.9 (11.0)	1.00	0.67
Gender Female Male	43 (54.4) 36 (45.6)	48 (60.8) 31 (39.2)	21 (53.8) 18 (46.2)	24 (61.5) 15 (38.5)	22 (55.0) 18 (45.0)	24 (60.0) 16 (40.0)	0.918	1.00
Ethnicity Hausa Igbo Yoruba Others	38 (48.1) 19 (24.1) 9 (11.4) 13 (16.5)	38 (48.1) 17 (21.5) 7 (8.9) 17 (21.5)	19 (48.7) 9 (23.1) 5 (12.8) 6 (15.4)	19 (48.7) 8 (20.5) 3 (7.7) 9 (23.1)	19 (47.5) 10 (25.0) 4 (10.0) 7 (17.5)	19 (47.5) 9 (22.5) 4 (10.0) 8 (20.0)	1.00	1.00
Marital status Married Non-married Divorced/separated. Widow/widower	46 (58.2) 15 (19.0) 3 (3.8) 15 (19.0)	65 (82.3) 11 (13.9) 3 (3.8)	22 (56.4) 8 (20.5) 1 (2.6) 8 (20.5)	32 (82.1) 5 (12.8) 2 (5.1)	24 (60.0) 7 (17.5) 2 (5.0) 7 (17.5)	33 (82.5) 6 (15.0) 1 (2.5)	0.94	1.00
Education High school or less College/university	51 (64.6) 28 (35.4)	42 (53.2) 37 (46.8)	26 (66.7) 13 (33.3)	20 (51.3) 19 (48.7)	25 (62.5) 15 (37.5)	22 (55.0) 18 (45.0)	0.699	0.823
Religion Islam Christianity Others	45 (57.0) 27 (34.2) 7 (8.9)	41 (51.9) 29 (36.7) 9 (11.4)	24 (61.5) 12 (30.8) 3 (7.7)	22 (56.4) 13 (33.3) 4 (10.3)	21 (52.5) 15 (37.5) 4 (10.0)	19 (47.5) 16 (40.0) 5 (12.5)	0.743	0.77
Occupation Working Not working	36 (45.6) 43 (54.4)	60 (75.9) 19 (24.1)	17 (43.6) 22 (56.4)	31 (79.5) 8 (20.5)	19 (47.5) 21 (42.5)	29 (72.5) 11 (27.5)	0.727	0.60
Monthly income >$500 (N200,000) <$500 (N200,000)	20 (25.3) 59 (74.7)	26 (32.9) 53 (67.1)	10 (25.6) 29 (74.4)	13 (33.3) 26 (66.7)	10 (25.0) 30 (75.0)	13 (32.5) 27 (67.5)	1.00	1.00
Cancer type Breast Colorectal Cervical Lung Prostrate Others	26 (32.9) 9 (11.4) 11 (13.9) 11 (13.9) 10 (12.7) 12 (15.2)		13 (33.3) 3 (7.7) 5 (12.8) 7 (17.9) 5 (12.8) 6 (15.4)		13 (32.5) 6 (15.0) 6 (15.0) 4 (10.0) 5 (12.5) 6 (15.0)		0.871	-
Stage of cancer Stage 1 Stage 2 Stage 3 Stage 4	3 (3.8) 32 (40.5) 43 (54.4) 1 (1.3)		1 (2.6) 17 (43.6) 20 (51.3) 1 (2.6)		2 (5.0) 15 (37.5) 23 (57.5)		0.729	-
Relationship with adults with cancer Son/daughter Spouse Parent Other family member i.e., Sibling, or cousin Friend		30 (38.0) 28 (35.4) 7 (8.9) 7 (8.9) 7 (8.9)		15 (38.5) 14 (35.9) 3 (7.7) 3 (7.7) 4 (10.3)		15 (37.5) 14 (35.0) 4 (10.0) 4 (10.0) 3 (7.5)	-	1.00

For all the variables there were no significant differences between groups which signifies non-bias selection/ grouping at baseline

**Table 3.** The results of MANOVA for the components of variables in adults with cancer.

Variable	Intervention group (*n* = 39)		Control group (*n* = 40)						
	Baseline (*n* = 39) M (SD)	4-week (*n* = 39) M (SD)	Baseline (*n* = 40) M (SD)	4-week (*n* = 40) M (SD)	Sum of squares	df	Mean of squares	F	*p*-value
Family/social support^a^ Significant other Family subscale Friend subscale Overall score	17.10 (6.84) 16.46 (7.20) 15.26 (7.43) 48.82 (18.97)	19.31 (4.95) 19.05 (5.08) 18.28 (4.70) 56.64 (13.67)	15.88 (4.87) 15.95 (5.07) 14.25 (5.27) 46.07 (12.5)	14.03 (3.35) 15.08 (4.17) 13.08 (3.85) 42.18 (9.51)	418.47 198.86 381.18 2,924.87	1 1 1 1	418.47 198.86 381.18 2,924.87	8.63 3.56 7.22 8.07	0.004* 0.063 0.009* 0.006*
Spiritual need^b^ Psychosocial subscale Spiritual subscale Religious subscale Overall score	13.15 (3.46) 32.77 (11.74) 13.51 (4.33) 59.44 (18.10)	12.54 (2.23) 25.92 (5.90) 12.21 (3.53) 50.67 (9.68)	12.15 (2.13) 30.98 (8.33) 13.38 (2.32) 56.50 (11.4)	14.05 (1.78) 34.13 (6.72) 14.58 (2.31) 62.75 (8.38)	2.55 405.39 49.19 826.16	1 1 1 1	2.55 405.39 49.19 826.16	0.26 3.40 2.61 3.03	0.612 0.069 0.110 0.086
Information need^c^	20.10 (8.38)	15.97 (6.15)	19.53 (3.61)	21.90 (3.25)	282.40	1	282.40	4.86	0.030*
Cancer health Literacy^d^	2.33 (1.61)	3.72 (1.08)	2.83 (1.08)	2.65 (0.98)	3.28	1	3.28	1.23	0.272
Quality of life^e^ Physical wellbeing Social wellbeing Emotional wellbeing Functional wellbeing Spiritual wellbeing Overall score	17.23 (7.12) 13.44 (5.56) 17.05 (4.09) 5.38 (5.35) 19.18 (10.15) 72.38 (12.05)	12.26 (4.59) 18.92 (4.23) 15.59 (3.31) 17.46 (3.73) 24.18 (8.65) 88.41 (11.80)	16.18 (4.98) 13.40 (3.74) 14.50 (2.33) 8.35 (4.11) 21.00 (6.35) 73.65 (9.09)	17.65 (4.35) 13.00 (3.14) 15.63 (2.34) 7.60 (3.24) 20.78 (6.07) 74.65 (8.36)	177.10 350.60 62.50 469.55 24.77 1,597.47	1 1 1 1 1 1	177.10 350.60 62.50 469.55 24.77 1,597.47	3.35 10.36 3.53 19.51 0.23 9.21	0.071 0.002* 0.064 0.000* 0.637 0.003*

Note: ^a^Multidimensional Scale of Perceived Social Support; ^b^Spiritual Needs Assessment for Patients; ^c^Comprehensive Needs Assessment Tool for Cancer (Subscale); ^d^Cancer Health Literacy; ^e^Functional Assessment of Chronic Illness Therapy-Spirituality; SD: standard deviation, F: MANOVA, dfh: degrees of freedom for the hypothesis, dfe: degrees of freedom for error, df: degrees of freedom, *equal to 5% (p < .05)

**Table 4.** The results of MANOVA for the components of variables in family caregivers.

Variable	Intervention group (*n* = 39)		Control group (*n* = 40)						
	Baseline (*n* = 39) M (SD)	4-week (*n* = 39) M (SD)	Baseline (*n* = 40) M (SD)	4-week (*n* = 40) M (SD)	Sum of squares	df	Mean of squares	*F*	*p*-value
Family/social support^a^ Significant other Family subscale Friend subscale Overall score	17.13 (6.31) 17.28 (6.24) 14.92 (6.60) 49.33 (17.9)	17.85 (5.65) 17.85 (5.42) 16.59 (5.11) 52.28 (15.23)	16.78 (5.30) 16.68 (5.72) 14.93 (5.42) 48.38 (14.8)	15.30 (4.11) 15.23 (4.62) 13.70 (4.27) 44.23 (11.56)	82.99 102.90 82.34 802.48	1 1 1 1	82.99 102.89 82.34 802.48	1.57 1.79 1.57 1.92	0.214 0.184 0.214 0.170
Spiritual need^b^ Psychosocial subscale Spiritual subscale Religious subscale Overall score	11.74 (2.68) 29.56 (10.10) 13.64 (4.13) 54.95 (15.6)	11.54 (2.04) 25.15 (5.64) 13.64 (4.13) 50.33 (10.04)	11.93 (2.45) 29.78 (7.71) 13.20 (2.96) 54.90 (11.4)	12.40 (2.21) 32.65 (6.37) 14.05 (2.46) 59.10 (9.13)	10.74 586.47 0.010 750.41	1 1 1 1	10.74 586.47 0.010 750.41	1.11 5.91 0.000 2.99	0.295 0.017* 0.983 0.088
Information need^c^	13.95 (5.67)	10.54 (3.71)	14.35 (4.59)	17.13 (7.36)	482.12	1	482.12	9.39	0.003*
Cancer health Literacy^d^	3.00 (1.49)	3.74 (1.07)	2.60 (1.66)	2.53 (1.71)	25.87	1	25.87	6.04	0.016*
Quality of life^e^ Physical wellbeing Social wellbeing Emotional wellbeing Functional wellbeing Spiritual wellbeing Overall score	7.33 (6.80) 16.15 (6.36) 16.97 (4.16) 11.51 (5.35) 25.18 (8.64) 77.15 (13.88)	6.85 (4.74) 19.28 (3.95) 14.36 (3.10) 17.95 (2.94) 29.82 (6.84) 88.26 (9.63)	8.80 (4.72) 16.05 (4.17) 15.03 (2.85) 11.93 (5.24) 24.63 (7.24) 76.43 (12.4)	11.83 (5.07) 14.93 (3.45) 16.33 (2.82) 10.95 (4.48) 23.55 (6.28) 77.58 (10.58)	410.19 196.48 0.003 428.33 459.91 1,285.46	1 1 1 1 1 1	410.19 196.48 0.003 428.33 459.91 1,285.46	7.84 5.09 0.000 11.80 4.94 5.39	0.006* 0.027* 0.990 0.001* 0.029* 0.023*

Note: ^a^Multidimensional Scale of Perceived Social Support; ^b^Spiritual Needs Assessment for Caregivers; ^c^Comprehensive Needs Assessment Tool for Family Caregiver (Subscale); ^d^Cancer Health Literacy; ^e^Functional Assessment of Chronic Illness Therapy-Spirituality; SD: standard deviation, F: MANOVA, dfh: degrees of freedom for the hypothesis, dfe: degrees of freedom for error, df: degrees of freedom, *equal to 5% (p < .05)

## Appendix: sample checklist

### The TIDieR (Template for Intervention Description and Replication) Checklist*:

Information to include when describing an intervention and the location of the information

**Table:**

Item number	Item	Where located **	
		Primary paper (page or appendix number)	Other ^†^ (details)
	**BRIEF NAME**		
**1.**	Provide the name or a phrase that describes the intervention.	_____________	_____________
	**WHY**		
**2.**	Describe any rationale, theory, or goal of the elements essential to the intervention.	_____________	_____________
	**WHAT**		
**3.**	Materials: Describe any physical or informational materials used in the intervention, including those provided to participants or used in intervention delivery or in training of intervention providers. Provide information on where the materials can be accessed (e.g. online appendix, URL).	_____________	_____________
**4.**	Procedures: Describe each of the procedures, activities, and/or processes used in the intervention, including any enabling or support activities.	_____________	_____________
	**WHO PROVIDED**		
**5.**	For each category of intervention provider (e.g. psychologist, nursing assistant), describe their expertise, background and any specific training given.	____________	_____________
	**HOW**		
**6.**	Describe the modes of delivery (e.g. face-to-face or by some other mechanism, such as internet or telephone) of the intervention and whether it was provided individually or in a group.	____________	_____________
	**WHERE**		
**7.**	Describe the type(s) of location(s) where the intervention occurred, including any necessary infrastructure or relevant features.	_____________	_____________
	**WHEN and HOW MUCH**		
**8.**	Describe the number of times the intervention was delivered and over what period of time including the number of sessions, their schedule, and their duration, intensity or dose.	____________	_____________
	**TAILORING**		
**9.**	If the intervention was planned to be personalised, titrated or adapted, then describe what, why, when, and how.	_____________	_____________
	**MODIFICATIONS**		
**10.^ǂ^**	If the intervention was modified during the course of the study, describe the changes (what, why, when, and how).	_____________	_____________
	**HOW WELL**		
**11.**	Planned: If intervention adherence or fidelity was assessed, describe how and by whom, and if any strategies were used to maintain or improve fidelity, describe them.	_____________	_____________
**12.^ǂ^**	Actual: If intervention adherence or fidelity was assessed, describe the extent to which the intervention was delivered as planned.	_____________	_____________

** **Authors** - use N/A if an item is not applicable for the intervention being described. **Reviewers** – use ‘?’ if information about the element is not reported/not sufficiently reported.

† If the information is not provided in the primary paper, give details of where this information is available. This may include locations such as a published protocol or other published papers (provide citation details) or a website (provide the URL).

ǂ If completing the TIDieR checklist for a protocol, these items are not relevant to the protocol and cannot be described until the study is complete.

* We strongly recommend using this checklist in conjunction with the TIDieR guide (see BMJ 2014;348:g1687) which contains an explanation and elaboration for each item.

* The focus of TIDieR is on reporting details of the intervention elements (and where relevant, comparison elements) of a study. Other elements and methodological features of studies are covered by other reporting statements and checklists and have not been duplicated as part of the TIDieR checklist. When a **randomised trial** is being reported, the TIDieR checklist should be used in conjunction with the CONSORT statement (see www.consort-statement.org) as an extension of **Item 5 of the CONSORT 2010 Statement.** When a** clinical trial protocol** is being reported, the TIDieR checklist should be used in conjunction with the SPIRIT statement as an extension of **Item 11 of the SPIRIT 2013 Statement** (see www.spirit-statement.org). For alternate study designs, TIDieR can be used in conjunction with the appropriate checklist for that study design (see www.equator-network.org).
